# A photoimmune synergizing RNA interference technology for highly effective antitumor treatment

**DOI:** 10.1002/smo2.70095

**Published:** 2026-08-23

**Authors:** Xiang Xia, Bingbing Wu, Xueze Zhao, Wen Sun, Jianjun Du, Jiangli Fan, Xiaojun Peng

**Affiliations:** ^1^ State Key Laboratory of Fine Chemicals Dalian University of Technology Dalian China; ^2^ The Key Laboratory of Laboratory Medicine Ministry of Education School of Laboratory Medicine and Life Sciences Wenzhou Medical University Wenzhou China

**Keywords:** phenothiazine cationic photosensitizer, photo‐crosslinking‐mediated RNAi therapy, photoimmunotherapy, pyroptosis, tumor treatment

## Abstract

Photoimmunotherapy and RNA interference (RNAi) hold great potential for tumor therapy but exhibit limited efficacy as monotherapies. In this work, we report a red‐light‐activated combinatorial therapy that integrates photodynamic immunotherapy (PIT) with RNAi via a rationally designed photosensitizer, FNBS, developed on the basis of our previously reported advanced photosensitizer platform, HNBS. Upon cellular uptake and following red light irradiation, FNBS induces pyroptosis through the activation of nucleotide‐binding oligomerization domain‐like receptor thermal protein domain associated protein 3 inflammasome pathways, thereby enhancing antitumor immune responses and converting “cold” tumors into “hot” tumors. Meanwhile, ROS‐mediated oxidation of the furan moiety enables covalent RNA crosslinking and subsequent RNA degradation, thereby facilitating targeted RNAi. Owing to this dual mechanism, effective cancer cell elimination is achieved under normoxic (21% O_2_) and hypoxic (2% O_2_) conditions. FNBS has an IC_50_ as low as 80 nM. In addition, RNA sequencing analysis reveals that FNBS‐induced cell death is closely associated with RNA‐related pathways, including RNA metabolism and RNA degradation. Collectively, this work establishes a novel combinatorial therapy strategy in which photodynamic therapy, immunotherapy, and RNA interference are integrated within a single small‐molecule platform, providing a foundation for achieving more effective cancer treatment.

## Graphical abstract

We developed a red‐light‐activated small‐molecule photosensitizer FNBS, which enables simultaneous photodynamic immunotherapy and RNA interference. FNBS can induce pyroptosis, immunogenic cell death and RNA degradation, avoiding the carrier‐dependent delivery drawbacks of conventional RNAi therapy, converting cold tumors into hot tumors and significantly enhancing antitumor efficacy.

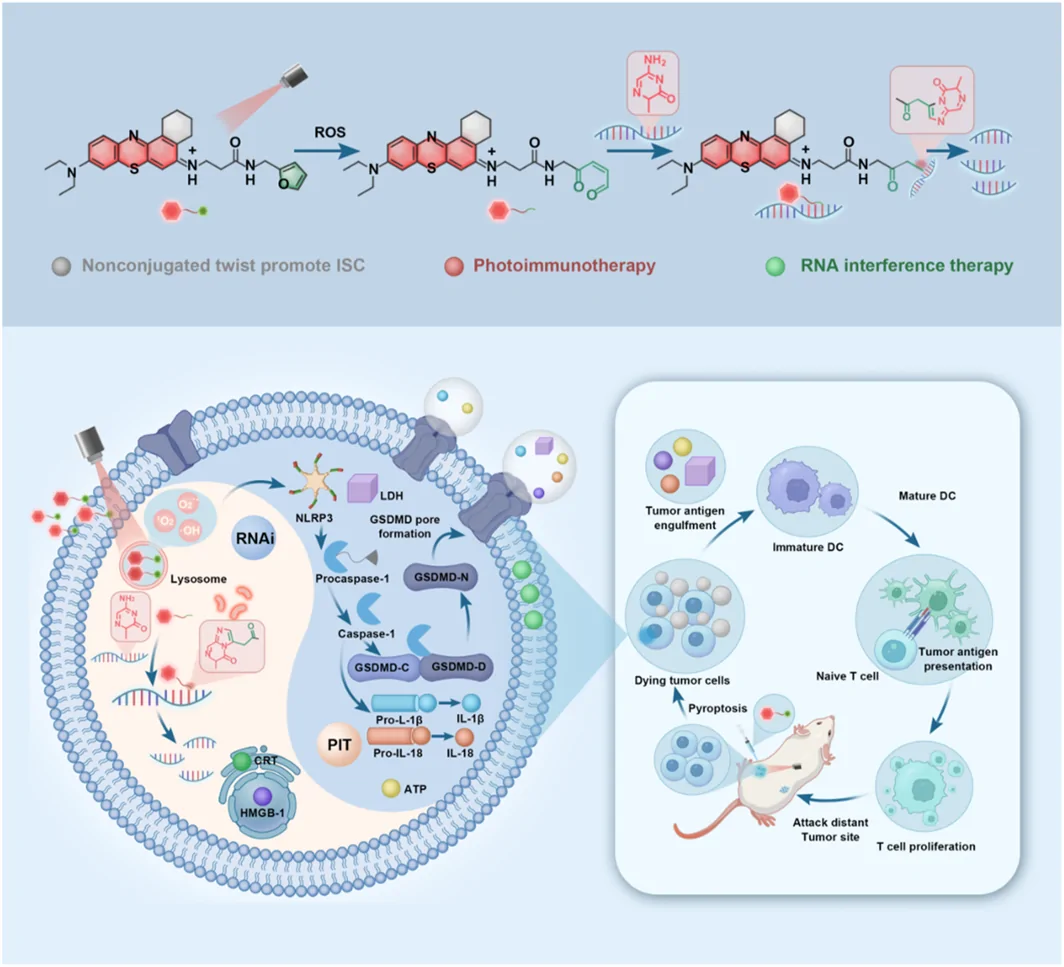

## 1 INTRODUCTION

Photoimmunotherapy (PIT), which combines photodynamic therapy (PDT) with immunotherapy (IMT), has emerged as a promising strategy for cancer treatment [1, 2, 3]. In its classical form, PIT relies on conjugating photosensitizers to tumor‐targeting antibodies [4]. Upon light irradiation, the photosensitizer generates reactive oxygen species (ROS), which directly kill tumor cells while simultaneously triggering immunogenic cell death (ICD) [5, 6]. Through ICD, tumor‐associated antigens and damage‐associated molecular patterns (DAMPs) are released, leading to the activation of dendritic cells (DCs) and cytotoxic T cells and, ultimately, remodeling of the immunosuppressive tumor microenvironment [7, 8]. By combining the localized cytotoxicity of PDT with the systemic immune modulation of IMT, PIT enhances antitumor efficacy and addresses key limitations of single‐modal PDT or IMT [9, 10, 11]. Despite these advantages, however, the therapeutic efficacy of PIT remains constrained by the relatively mild inflammatory response associated with ICD [12, 13]. This limitation is particularly evident in so‐called “cold” tumors, which are characterized by poor immune cell infiltration [14]. In this context, pyroptosis, a pro‐inflammatory form of programmed cell death mediated by inflammasomes, has attracted increasing attention [15, 16]. Pyroptosis involves cleavage of gasdermin D (GSDMD), resulting in membrane pore formation and the massive release of pro‐inflammatory cytokines, including IL‐1β and IL‐18, thereby eliciting a stronger systemic immune response than ICD [17, 18]. Consequently, the induction of pyroptosis as a new mode of PIT holds considerable potential for overcoming the insufficient immunogenicity of classical PIT.

On the other hand, small interfering RNA (siRNA), a type of RNA interference (RNAi) technology, is a double‐stranded non‐coding RNA molecule that can mediate the sequence‐specific degradation of target mRNA, thereby achiving post‐transcriptional gene silencing and effectively inhibiting protein synthesis [19, 20]. Therefore, siRNA has great potential in treating various life‐threatening diseases such as cancers [21, 22, 23]. However, owing to its hydrophilic and polyanionic properties, naked siRNA is unable to cross the cell membrane and is easily degraded by ribonucleases, resulting in minimal therapeutic activity [24, 25]. Even carrier‐mediated delivery systems are generally facilitating cellular entry via endocytosis and entrapped in endosomes, the endosomes will fuse with the lysosomes, leading to reduced or even no release of siRNA in the cytosol to induce gene silence. It is noteworthy that under the influence of ROS, furan will undergo ring‐opening to form 4‐oxo‐enal, which further undergo Michael type addition reactions with cytosine, ultimately achieving nucleic acid cross‐linking and RNAi therapy [26, 27]. This strategy eliminates the need for preparing sequence‐specific siRNA fragments, enabling RNAi therapy for low cost and convenience [28]. Therefore, combining furan with photoimmunotherapy to develop a more efficient and universal RNAi‐photoimmunotherapy synergistic antitumor strategy is crucial for improving tumor therapeutic efficacy.

**Scheme 1:**
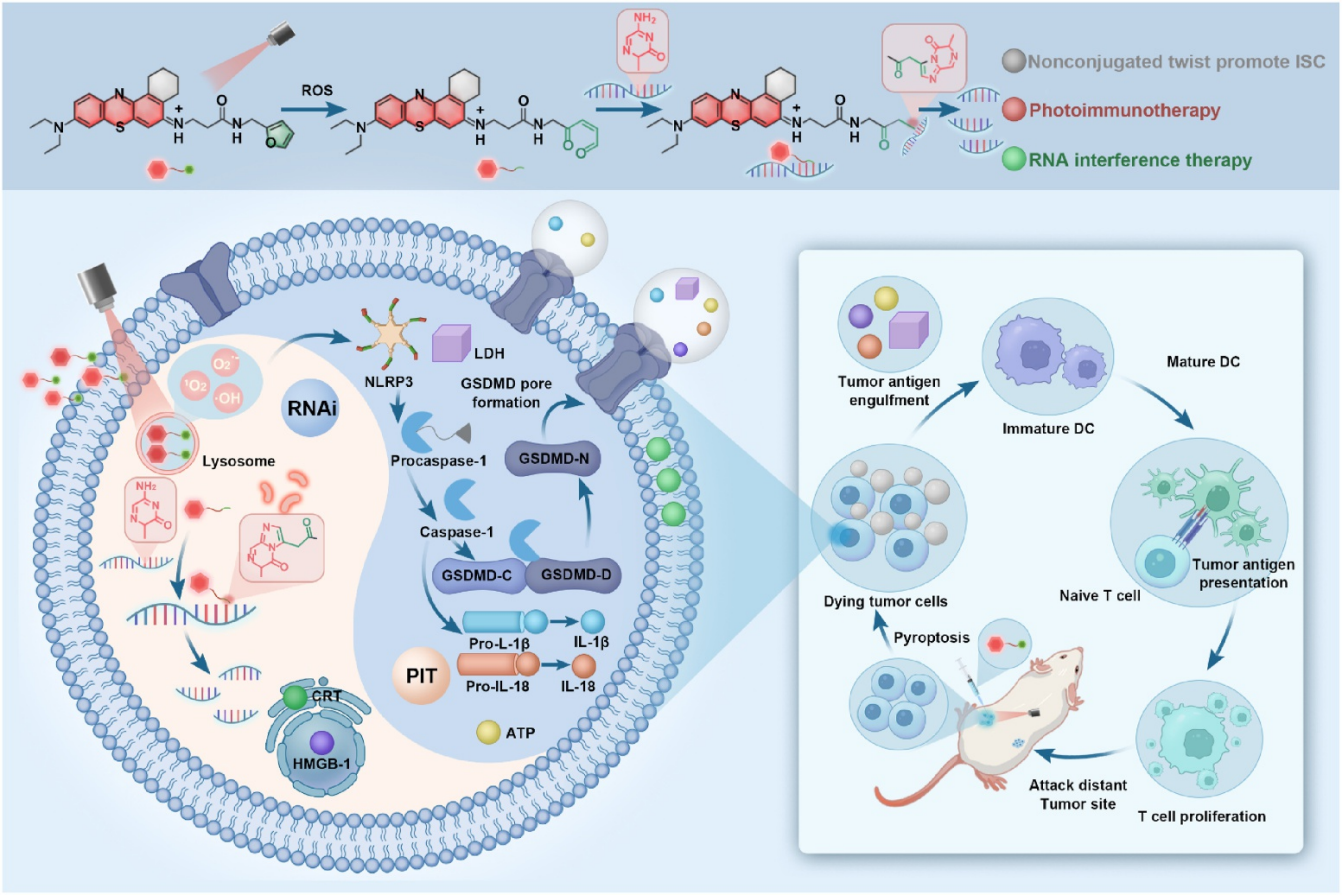
Schematic illustration of the photosensitizer for cancer that synergistically integrates photoimmunotherapy with RNA interference.

Currently, leveraging our previously reported photosensitizer platform [29], HNBS, together with the furan‐a functional group for RNAi therapy, we integrated PIT and furan‐mediate RNAi therapy into a small‐molecule photosensitizer (PS), named FNBS (Scheme 1). FNBS can possess a prominent capacity for ROS generation upon light irradiation. Thus, even after internalization into lysosomes, the photosensitive dyes are capable of lysosomal escape into the cytoplasm under light irradiation. Concomitantly, this process activates the NLRP3 inflammasome, triggers the release of caspase‐1‐dependent pro‐inflammatory cytokines, and ultimately culminates in pyroptosis and ICD. This process promotes the maturation of DCs, natural killer (NK) cells, and T cells, thereby enhancing the immune response and converting “cold” tumors into “hot” tumors. Meanwhile, ROS oxidizes the furan moiety in FNBS. Following lysosomal damage, the oxidized furan moiety binds to RNA in the cytosolic matrix and mediates RNA degradation under light irradiation. Therefore, the synergistic effect of PIT and photo‐crosslinking‐mediated RNAi therapy is achieved simultaneously.

## 2 RESULTS AND DISCUSSION

### 2.1 Design and synthesis

In our previous study, we reported an effective photosensitizer platform, HNBS, which can trigger pyroptosis under an ultra‐low light dose, thereby enabling PIT. To further improve tumor treatment outcomes, a photoactive functional group, furan, was incorporated, resulting in a new PS named FNBS. FNBS enables the simultaneous execution of PIT and RNAi therapy under red light irradiation. FNBS was synthesized through a four‐step process (Supporting Information S1: Figure S1). *N,N*‐Diethyl‐*p*‐phenylenediamine and 5,6,7,8‐tetrahydronaphthalen‐1‐amine were used as the starting materials. Intermediate 2 was first prepared by electrophilic aromatic substitution, followed by amide condensation to yield intermediate 3. Ultimately, the target compound FNBS was afforded via silver carbonate‐catalyzed cyclization with Bunte salt. The chemical structures of the target compounds and intermediates were characterized by nuclear magnetic resonance spectroscopy and mass spectrometry (Supporting Information S1: Figures S13–23).

### 2.2 Photophysical characterization and red‐light‐triggered RNA binding behavior in vitro

**FIGURE 1:**
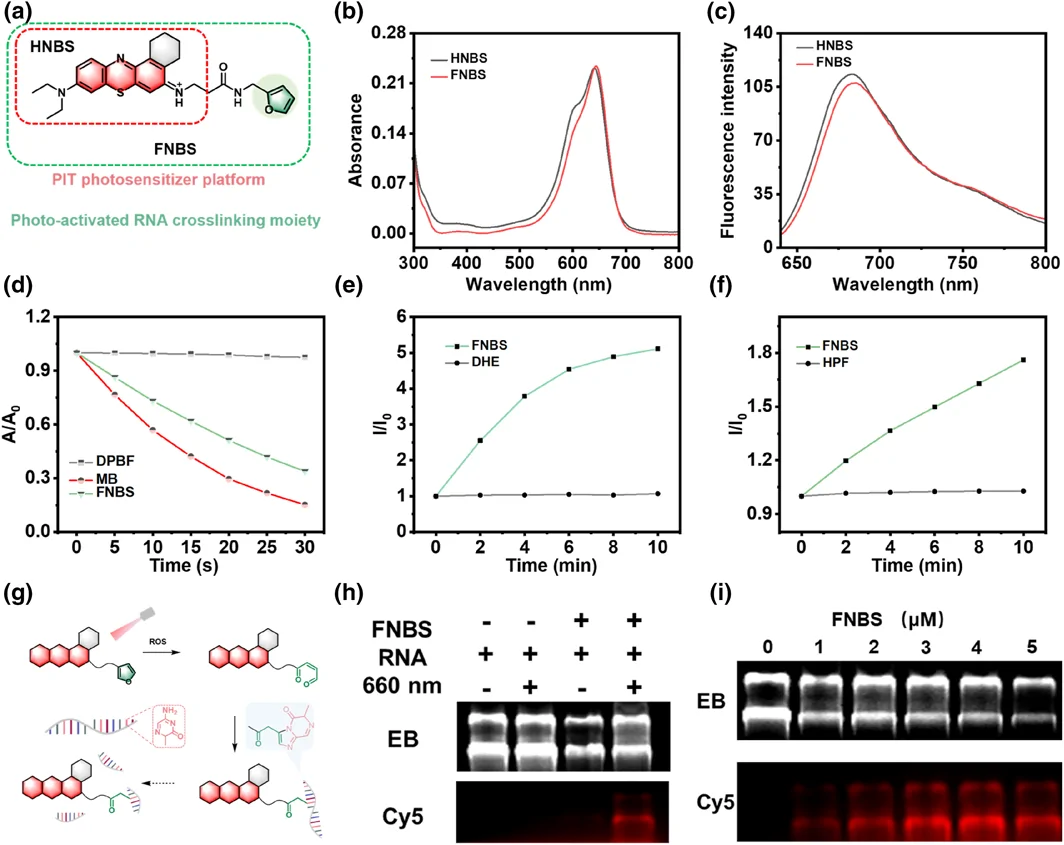
Photophysical properties and red light triger RNA binding Behavior of FNBS in vitro. (a) Chemical structure of HNBS and FNBS. (b) Absorption spectra of HNBS and FNBS in PBS. (c) Emission spectra of HNBS and FNBS in PBS. (d) Normalized changes in absorbance of DPBF at 411 nm induced by FNBS, with MB as the reference, under 660 nm light irradiation. (e) Superoxide anion generation by FNBS measured with DHE under 660 nm LED light irradiation. (f) Hydroxyl radical generation by FNBS measured with HPF under 660 nm LED light irradiation. (g) Schematic illustration of the mechanism of covalent RNA modification via cyclization between FNBS and RNA. (h) RNA agarose gel electrophoresis images of different treatment groups acquired under EB (546 nm) and Cy5 (660 nm) excitation. (i) RNA agarose gel electrophoresis images of FNBS at different RNA concentrations.

In order to evaluate whether the introduction of the photoactivatable furan group affects the photosensitizing performance of photosensitizers (Figure 1a), we compared the photophysical properties of FNBS and HNBS. First, the photophysical properties of FNBS and HNBS (Supporting Information S1: Figure S1) were evaluated. The absorption and emission spectra (Figure 1b,c and Supporting Information S1: Figure S2) of HNBS and FNBS are similar, indicating that incorporation of the furan group does not significantly alter the photophysical properties of HNBS. Second, the ROS production capacity of FNBS was subsequently assessed [30, 31]. 1,3‐diphenylisobenzofuran (DPBF) was used as a singlet oxygen (^1^O_2_) indicator to evaluate ^1^O_2_ generation by FNBS. As shown in Figure 1d and Supporting Information S1: Figure S3, FNBS exhibits slightly lower ^1^O_2_ generation yield (36.7%) relative to HNBS (42.0%). This discrepancy may result from ROS consumption associated with the ring‐opening reaction of furan. In addition, the generation of superoxide anion (O_2_•^−^) and hydroxyl radical (•OH) was evaluated using dihydroethidium (DHE) and hydroxyphenyl fluorescein (HPF) as respective indicators (Figure 1e,f and Supporting Information S1: Figures S4) [32, 33], demonstrating that FNBS retains the excellent photophysical properties and ROS‐generating ability of HNBS.

After confirming that FNBS retained the highly efficient photosensitizing capacity of photosensitizers, we further investigated its RNA‐binding ability in vitro. Furan moiety in FNBS is known to mediate crosslinking with RNA nucleotides (Figure 1g). Specifically, light irradiation of FNBS triggers ROS production, which enables RNA labeling; prolonged light exposure, however, induces RNA degradation, thereby facilitating the subsequent implementation of RNAi therapy. To verify these functions, we evaluated the capacity of red‐light‐activated FNBS to form covalent bonds with RNA in vitro. For this assay, RNA was extracted from 4T1 cells using an RNA extraction kit, and the extracted RNA was then incubated with FNBS under either 660 nm LED irradiation or dark conditions. As depicted in Figure 1h, agarose gel electrophoresis images of different treatment groups were acquired under excitation at 546 nm (EB) and 660 nm (FNBS). No red fluorescence signal was observed in the Cy5 channel for the RNA group, the RNA with light irradiation group, or the photosensitizer and RNA without light irradiation group. In contrast, red fluorescence appeared when RNA was combined with both the photosensitizer and light irradiation.

These results demonstrate that RNA photo‐crosslinking requires the simultaneous presence of the photosensitizer, light, and RNA. In addition, crosslinked gel electrophoresis experiments were conducted using the photosensitizer and RNA at different concentration gradients. The fluorescence intensity initially increased and subsequently decreased with increasing FNBS concentration (Figure 1i), which is likely attributable to RNA degradation induced by excessive FNBS exposure. These results indicate that FNBS retains the excellent properties of reported photosensitizers, and achieves RNA photo‐crosslinking and triggered degradation via its incorporated furan moieties.

### 2.3 Photoinduced generation of ROS in cancer cells

**FIGURE 2:**
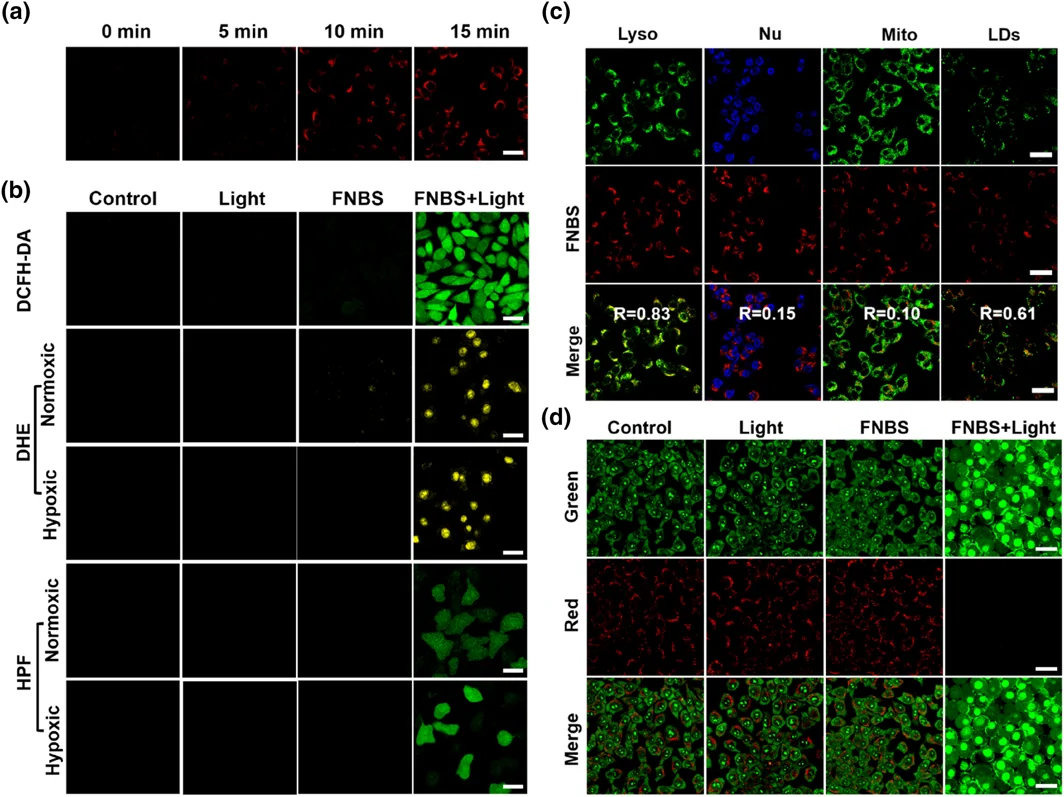
Fluorescence imaging of FNBS in 4T1 cells. (a) Cellular uptake of FNBS in 4T1 cells at different incubation times. Scale bar: 30 μm (b) Intracellular total reactive oxygen species (ROS), •OH, and O_2_•^−^ generation induced by different treatments, detected by DCFH‐DA, HPF, and DHE staining. (c) Subcellular colocalization images of FNBS with Lyso‐Tracker Green (Lyso), Hoechst 33342 (Nu), Mito‐Tracker Green (Mito), and lipid droplets (LDs) in 4T1 cells; P denotes the Pearson correlation coefficient (scale bar: 30 μm). (d) Fluorescence images of Acridine orange (AO) staining used to evaluate lysosomal integrity in 4T1 cells with various treatments.

Inspired by the excellent ROS‐generation capability of FNBS in solution, its cellular uptake and intracellular ROS production were further investigated in 4T1 cells. As shown in Figure 2a, no red fluorescence signal was observed in the absence of FNBS. Upon incubation with FNBS, the intracellular red fluorescence intensity increased progressively and reached a maximum within 15 min, indicating the rapid internalization of FNBS by tumor cells. Intracellular ROS generation was subsequently evaluated using the DCFH‐DA probe (Figure 2b). Negligible fluorescence was detected in the control, control + light, and FNBS‐only groups, whereas the FNBS + light group showed a pronounced green fluorescence signal, confirming efficient ROS production upon photoactivation. To further identify the specific ROS species, DHE and HPF were employed to detect O_2_•^−^ and •OH, respectively, under both normoxic and hypoxic conditions. No detectable fluorescence signal was observed in the control, control + light, or FNBS‐only groups regardless of oxygen availability. In contrast, only the photosensitizer + light group displayed strong fluorescence signals were exclusively observed in the FNBS + light group under both conditions, indicating that FNBS is capable of generating ROS efficiently under both normoxic and hypoxic conditions upon light irradiation.

To elucidate the subcellular localization of FNBS, subcellular colocalization analysis was performed. Fluorescence imaging of FNBS co‐incubated with commercial sub‐organelle staining dyes revealed pronounced colocalization with LysoTracker Green (Figure 2c), yielding a Pearson correlation coefficient of 0.83 and indicating strong lysosomal targeting. However, following light exposure, FNBS undergoes lysosomal escape, as illustrated in Figure 2d. Acridine orange (AO) was employed as an imaging probe. In intact lysosomes, AO emits red fluorescence; following lysosomal rupture, AO redistributes to the nucleus or cytoplasm and emits green fluorescence. In the control, control + light, and FNBS‐only groups, intense red fluorescence was consistently observed, indicating preservation of lysosomal integrity. In contrast, in the FNBS + light irradiation group, red fluorescence was substantially diminished, consistent with lysosomal disruption. In addition, to verify whether FNBS induces damage to intracellular RNA, RNA content in cells from different treatment groups was quantified using an RNA extraction kit (Supporting Information S1: Table S1). Notably, the RNA content of the control group was 390 ± 8.2 ng/μL, while that of the FNBS group was 401 ± 9.8 ng/μL, both of which were substantially higher than that of the FNBS + light group (130 ± 5.9 ng/μL). These results confirm that light irradiation enables FNBS to undergo lysosomal escape, re‐enter the cytoplasm, bind to RNA, and mediate its crosslinking and degradation.

### 2.4 Evaluation of cytotoxicity in vitro

**FIGURE 3:**
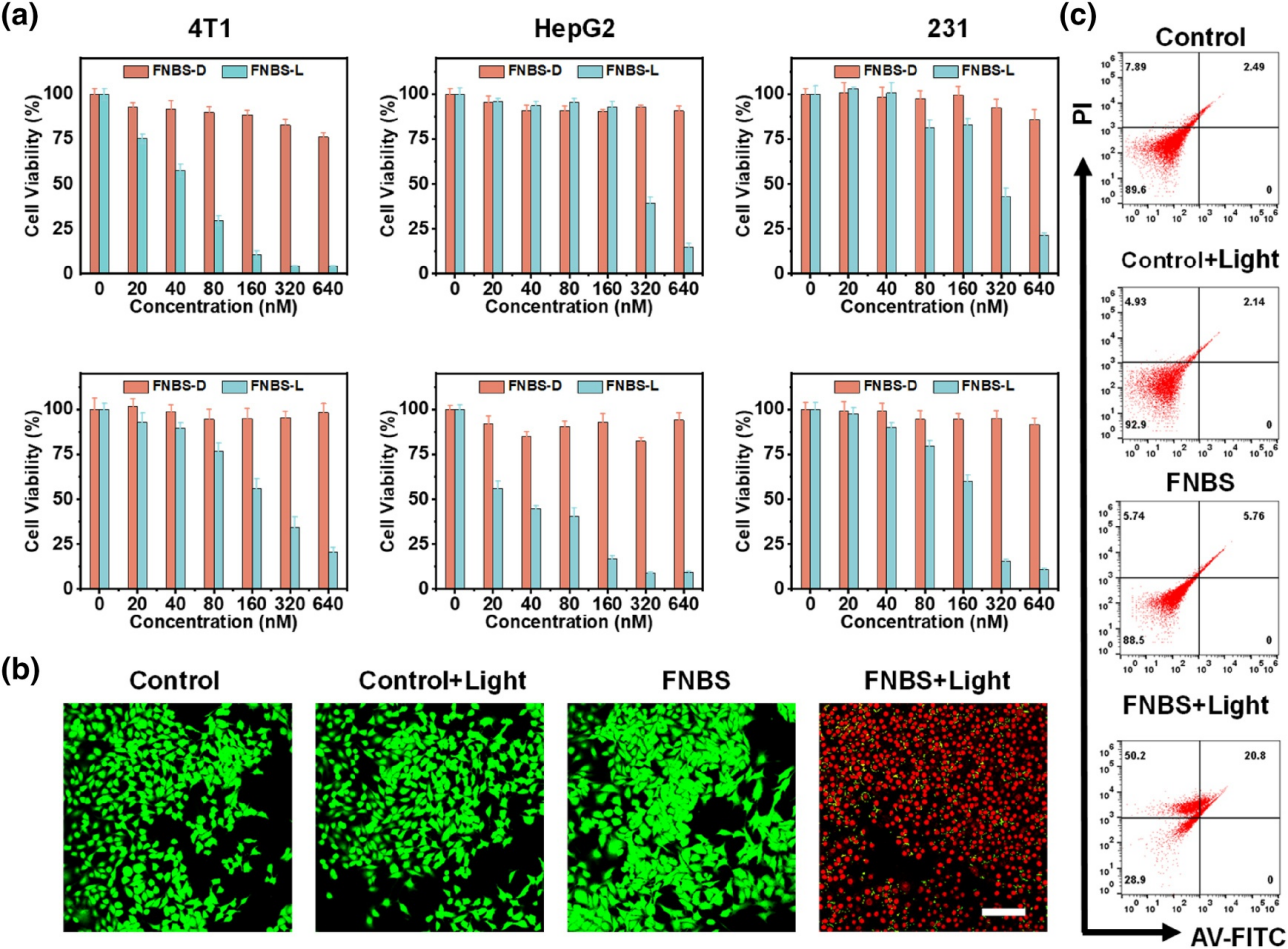
In vitro anti ‐cancer properties of FNBS. (a) Cell viability of 4T1, HepG2, and MDA‐MB‐231 cells after treatment with various concentrations of FNBS in the presence or absence of light irradiation (660 nm, 0.45 J cm^−2^) under normoxic (top panel) and hypoxic (bottom panel) conditions. (b) Live/dead staining of 4T1 cells following different treatments. (c) Apoptotic and necrotic cell death in different groups analyzed by flow cytometry.

A standard MTT assay was performed using HepG2, 4T1, and MDA‐MB‐231 cells to evaluate the cytotoxicity of FNBS (Figure 3a,b). In the absence of light irradiation, FNBS exhibited low cytotoxicity across all tumor cell lines under both normoxic and hypoxic conditions, with cell survival rates exceeding 90%. In contrast, upon light irradiation (0.45 J cm^−2^), a marked increase in cytotoxicity was observed under both normoxic and hypoxic conditions. The half‐maximal inhibitory concentrations (IC_50_) of FNBS in HepG2, 4T1, and MCF7 cells were all below 200 nM under both conditions. Next, the phototherapeutic effect of FNBS was examined. Calcein‐AM staining (live cells, green channel) and propidium iodide (PI) staining (dead cells, red channel) were used to visualize cell viability (Figure 3c) [34]. Bright green fluorescence was observed in the control, control + light, and FNBS‐only groups, indicating that most cells remained viable. By contrast, the FNBS + light group exhibited intense red fluorescence with almost no green signal, demonstrating the good biocompatibility of FNBS in the dark and its pronounced photocytotoxicity upon light irradiation. In addition, the cell death mechanism induced by FNBS, including apoptosis and necrosis, was evaluated by flow cytometry using Annexin V‐FITC and PI staining (Figure 3d). Consistent with the MTT assay results, the control, control + light, and FNBS‐only groups showed cell survival rates exceeding 88%. In contrast, the FNBS + light irradiation group displayed a markedly reduced cell survival rate of 28.9%. Furthermore, more than half of the cells in this group underwent necrotic cell death after treatment, which is consistent with previous reports indicating that pyroptosis predominantly results in necrotic cell death.

### 2.5 Photoinduced pyroptosis and ICD induced by FNBS

**FIGURE 4:**
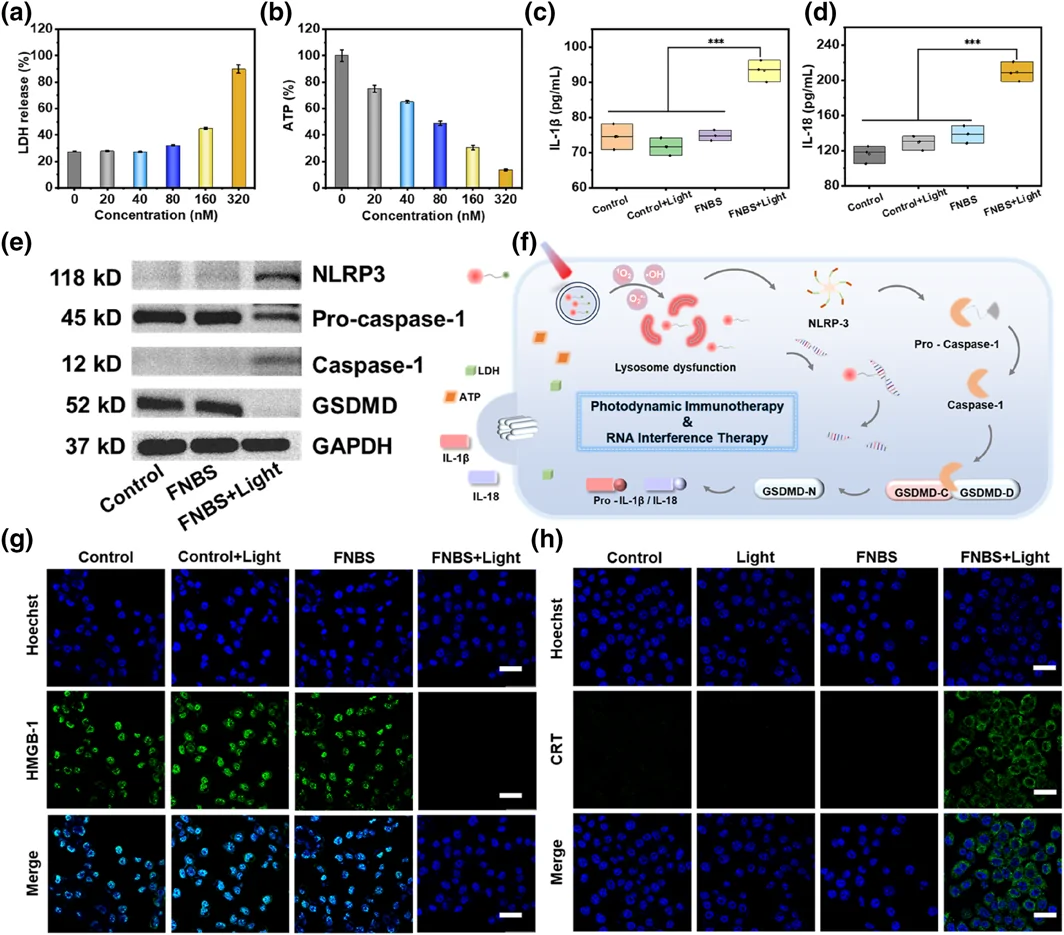
Pyroptosis and immunogenic cell death (ICD) mechanism of FNBS. (a) and (b) Release of Lactate dehydrogenase (LDH) and intracellular ATP from 4T1 cells after different treatments. (c) and (d) Release of IL‐1β and IL‐18 from 4T1 cells after different treatments (*n* = 3). (e) Detection of pyroptosis‐related proteins (NLRP3, GSDMD, Caspase‐1) by Western blot analysis. (f) Schematic illustration of photodynamic immunotherapy and RNA interference therapy mediated by FNBS. (g) and (h) Immunofluorescence staining of CRT and high‐mobility group box 1 (HMGB‐1) (λex = 488 nm, λem = 500–540 nm) in 4T1 cells after different treatments, with nuclear staining using Hoechst (blue channel, λex = 405 nm, λem = 440–480 nm; scale bar: 40 μm). Mean ± SD; one‐way ANOVA, *n* = 3; ***, *p* < 0.001.

Pyroptosis, characterized by cell swelling, membrane rupture, and the release of intracellular contents, triggers ICD and activates tumor immunogenicity [35, 36, 37]. To elucidate the cell death modality induced by FNBS, multiple biochemical and molecular markers related to pyroptosis and ICD were systematically evaluated in 4T1 cells. Lactate dehydrogenase (LDH) and adenosine triphosphate (ATP) release were quantified using commercial assay kits, while the secretion of interleukin‐18 (IL‐18) and interleukin‐1β (IL‐1β) was determined by enzyme‐linked immunosorbent assay (ELISA). In parallel, the protein expression levels of nucleotide‐binding oligomerization domain‐like receptor protein 3 (NLRP3), gasdermin D (GSDMD), and Caspase‐1 were analyzed by Western blotting. As shown in Figure 4a, FNBS treatment under light irradiation induced a concentration‐dependent increase in LDH levels in the culture medium, indicating enhanced membrane permeabilization and LDH efflux, a hallmark of pyroptotic cell death. Consistently, ATP release exhibited a similar concentration‐dependent trend (Figure 4b), supporting the induction of ICD‐associated signals. In addition, ELISA analysis revealed that FNBS combined with light irradiation markedly increased the secretion of IL‐18 and IL‐1β by 1.9‐ and 1.3‐fold, respectively, compared with the control group (Supporting Information S1: Figure 4c,d), indicative of a robust inflammatory response within the tumor microenvironment.

In an attempt to delineate the signaling pathway involved in FNBS‐induced pyroptosis, we performed a Western blot analysis to evaluate the expression levels of NLRP3, GSDMD, and Caspase‐1 (Figure 4e and Supporting Information S1: Figure S5). The results demonstrated that NLRP3 expression was significantly elevated in the FNBS + light group, whereas no obvious changes were observed in the control or FNBS‐only groups. Concurrently, the levels of GSDMD and Caspase‐1 were also markedly increased upon photoactivation of FNBS, confirming activation of the NLRP3 inflammasome and its downstream pyroptotic machinery. Based on these results, a mechanistic pathway for FNBS‐induced pyroptosis is proposed (Figure 4f). Following cellular internalization, FNBS accumulates in lysosomes and generates ROS upon light exposure. ROS‐mediated damage to lysosomal membranes triggers oxidative processes involving furan, leading to lysosomal dysfunction. Furthermore, ROS activates NLRP3, resulting in the assembly of the NLRP3 inflammasome. Consequently, Caspase‐1 is cleaved and activated, which further processes GSDMD into its active fragment, N‐GSDMD. The formation of N‐GSDMD‐mediated membrane pores facilitates the release of inflammatory factors. Meanwhile, reactive intermediates generated during furan photooxidation undergo crosslinking reactions with nucleic acids, ultimately resulting in RNA degradation under prolonged light exposure.

The occurrence of ICD was further evaluated by immunofluorescence (IF) staining of calreticulin (CRT) and high‐mobility group box 1 (HMGB‐1) (Figure 4g,h). These molecules act as immune adjuvants that promote the recruitment and maturation of antigen‐presenting cells. In the FNBS + light group, extremely weak green fluorescence was observed in the nucleus, whereas bright green fluorescence was retained in the nuclei of the control, control + light, and FNBS groups, indicating translocation of HMGB‐1 from the nucleus to the extracellular space upon FNBS treatment with light irradiation. In addition, IF staining of CRT revealed bright fluorescence in the FNBS + light group, while no apparent green fluorescence was observed in other groups, suggesting light‐induced translocation of CRT from the endoplasmic reticulum to the cell membrane in the presence of FNBS. Collectively, these results demonstrate that FNBS induces pyroptosis and ICD, activates antitumor immune responses, and converts “cold” tumors into “hot” tumors.

### 2.6 RNA‐sequencing analysis of FNBS killing mechanism

**FIGURE 5:**
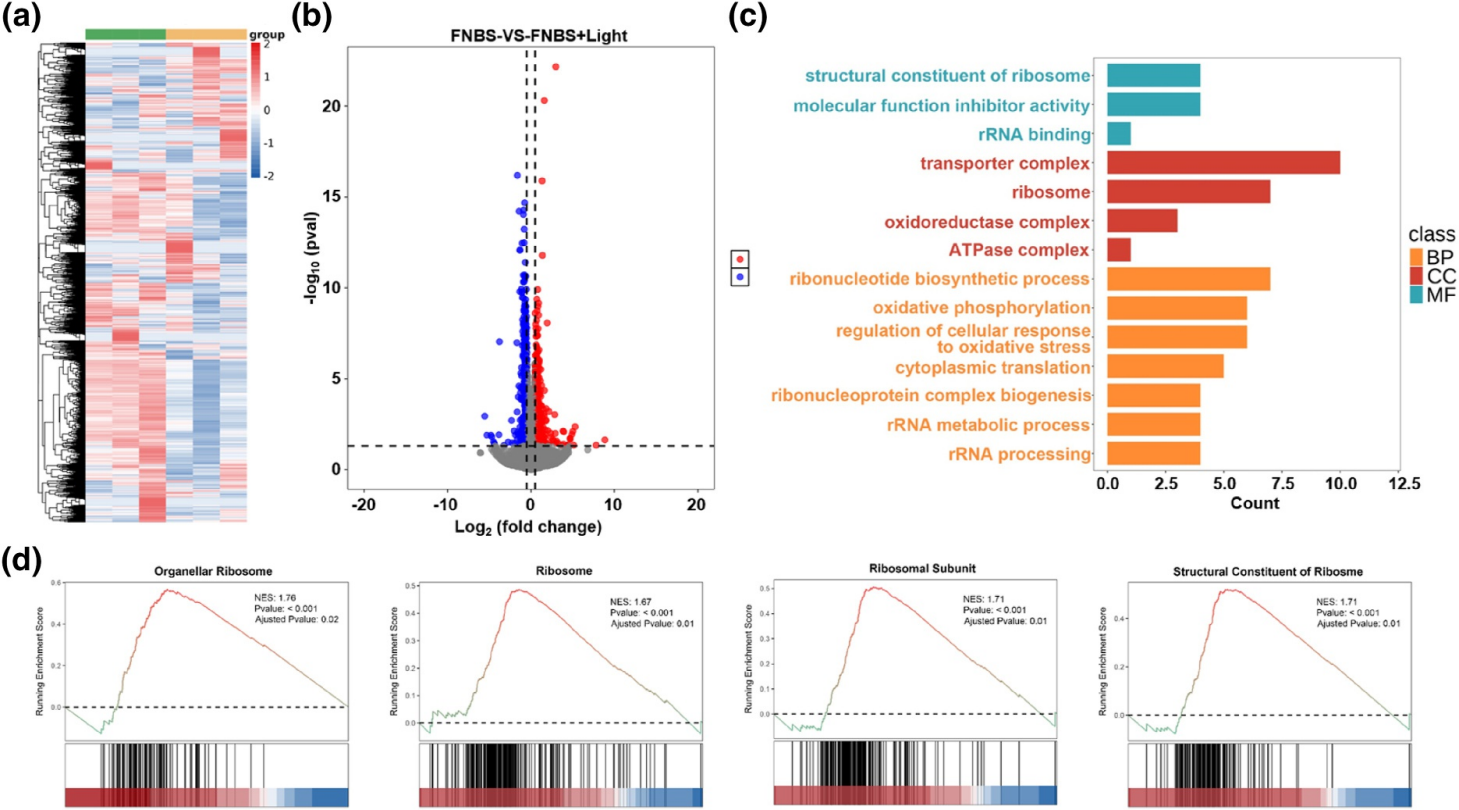
RNA‐seq and pathway enrichment analysis of 4T1 cells treated with FNBS in the presence or absence of light exposure. (a) Heat map showing differentially expressed genes (DEGs) in FNBS and FNBS + light samples (criteria: fold change ≥2, adjusted *p* value < 0.05). (b) Volcano plot comparing the gene expression levels of the FNBS and FNBS + light groups. (c) Gene Ontology (GO) enrichment analysis of genes whose expression levels were significantly altered following PDT. These genes are categorized as belonging to the biological process (BP), cellular component (CC), and molecular function (MF) classes. (d) Gene Set Enrichment Analysis (GSEA) analysis of significantly enriched pathways in FNBS + light‐treated cells. |NES| > 1, adjusted *p* value ≤ 0.05, and an FDR ≤25% were considered significant.

Following demonstration of the therapeutic efficacy of FNBS in inhibiting tumor cell proliferation under 660 nm LED light irradiation in vitro, we conducted RNA‐seq analysis to compare mRNA expression differences between the FNBS + light and FNBS groups. The overall aim of this analysis was to elucidate potential therapeutic mechanisms. Because FNBS treatment alone did not significantly affect cell viability, the analysis focused on identifying differentially expressed genes by comparing FNBS alone with FNBS under light irradiation. The experiment was independently repeated three times, demonstrating high reproducibility, with Pearson correlation coefficients ranging from 0.98 to 1.0 (Supporting Information S1: Figure S6). Initially, a heatmap was generated to visualize differential gene expression, comprising 8501 genes, in 4T1 cells treated with FNBS in the presence or absence of light irradiation (Figure 5a). As illustrated in the volcano plot (Figure 5b), relative to the FNBS group, 476 genes were upregulated and 637 genes were downregulated following light irradiation.

Subsequently, Gene Ontology analysis highlighted significant differences in 4T1 cells before and after PDT, particularly in pathways related to structural constituents of ribosome, transporter complex, ribosome, ribonucleotide biosynthetic process, and rRNA metabolic processes (Figure 5c). Furthermore, Gene Set Enrichment Analysis indicated pronounced changes in gene expression associated with organellar ribosomes, ribosome, ribosomal subunit, and structural constituent of ribosome (Figure 5d). Collectively, these findings strongly suggest that FNBS‐induced cell death is closely associated with RNA‐related pathways, including RNA metabolism and degradation, which aligns well with PDT‐mediated RNAi therapy.

### 2.7 Antitumor efficacy of FNBS in vivo

**FIGURE 6:**
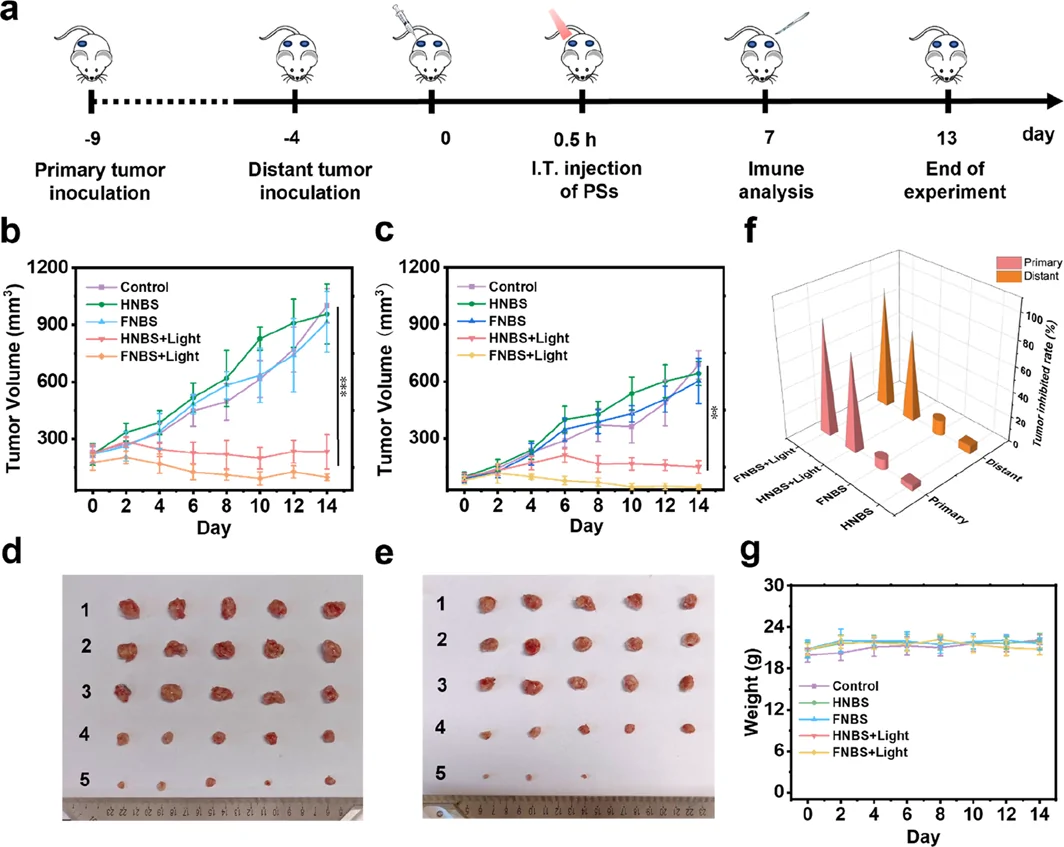
In vivo evaluation of tumor suppression in 4T1 subcutaneous bilateral tumor BALB/c mice. (a) Schematic illustration of treatment schedules for in vivo antitumor therapy. (b, c) Tumor volume change curves for primary and distant tumors in mice following different treatments. Photographs of primary (d) and distant (e) tumors excised from mice after PDT treatments (1. Control, 2. HNBS, 3. FNBS, 4. HNBS + light, 5. FNBS + light). (f) Tumor inhibition rates of primary and distant tumors. (g) Body weight changes of mice with different treatments. Mean ± SD; one‐way ANOVA, *n* = 5; **p < 0.01, ***, *p* < 0.001.

The in vivo antitumor efficacy of FNBS was evaluated using a bilateral subcutaneous tumor model established in Balb/C mice by inoculating 4T1 cells on both flanks with a 5‐day interval between injections (Figure 6a). The mice were randomly assigned to four treatment groups: control, HNBS, FNBS, HNBS + light, and FNBS + light. After intratumoral injection of FNBS, tumors were irradiated with 660 nm LED light for 5 minutes at 30 minutes post administration. As shown in Supporting Information S1: Figure S7, bright fluorescence signals were observed within the tumor upon injection, and fluorescence retention persisted for up to 72 hours. In contrast, HNBS exhibited a markedly shorter tumor retention time of approximately 6 h (Supporting Information S1: Figure S8), which is likely attributable to FNBS binding to cytoplasmic RNA, resulting in prolonged tumor retention.

In addition, the following irradiation of the primary tumor, the volumes of both primary and distant tumors were monitored throughout the treatment period. As shown in Figure 6b,c, no significant differences in tumor growth were observed among the control, control + light, and FNBS groups, indicating that light irradiation or FNBS alone produced no therapeutic effect. These observations were further supported by ex vivo tumor images (Figure 6d,e). In contrast, the FNBS + light group exhibited pronounced inhibition of both primary and distant tumors, with inhibition rates of 90.2% and 93.9%, respectively (Figure 6f), demonstrating that FNBS not only effectively induces PDT but also activates immune responses capable of suppressing distant tumor growth.

Moreover, no significant changes in body weight were observed among the treatment groups (Figure 6g), indicating good systemic tolerance. Hemolysis rates in all groups were below 5%, confirming the excellent blood compatibility of FNBS (Supporting Information S1: Figure S9). To further assess the in vivo performance and biocompatibility of FNBS, histological analysis using hematoxylin and eosin (H&E) staining was conducted on tumor tissues and major organs (Supporting Information S1: Figure S10), together with routine blood tests (Supporting Information S1: Figure S11). As shown in Figure S10, significant cellular damage was observed only in tumors from the FNBS + light group, whereas no apparent damage was detected in other groups. In addition, no pathological abnormalities were observed in the major organs, further confirming the good biocompatibility of FNBS. Routine blood analysis, including white and red blood cell counts, also revealed no notable differences among the treatment groups. Collectively, these results demonstrate that FNBS exhibits high in vivo antitumor efficacy under light irradiation together with excellent biocompatibility.

### 2.8 Pyroptosis‐mediated tumor microenvironment remodeling enhances antitumor immunity

The occurrence of ICD in cancer cells following pyroptosis is necessary for eliciting an antitumor immune response in vivo, as ICD promotes the release of DAMPs, including CRT and HMGB‐1, thereby promoting antitumor immunity. To verify whether FNBS‐based PDT induces pyroptosis‐associated ICD in vivo, immunofluorescence staining was performed on tumor tissues collected from mice subjected to different treatments. As shown in Supporting Information S1: Figure S12, bright fluorescence was observed for CRT, whereas HMGB‐1 fluorescence disappeared in the FNBS + light group, indicating that FNBS‐mediated PDT induces pyroptosis. Consistently, strong IF signals for Caspase‐1 and GSDMD were also detected, confirming that FNBS‐mediated PDT triggers pyroptosis‐associated ICD in vivo, in agreement with the in vitro findings.

**FIGURE 7:**
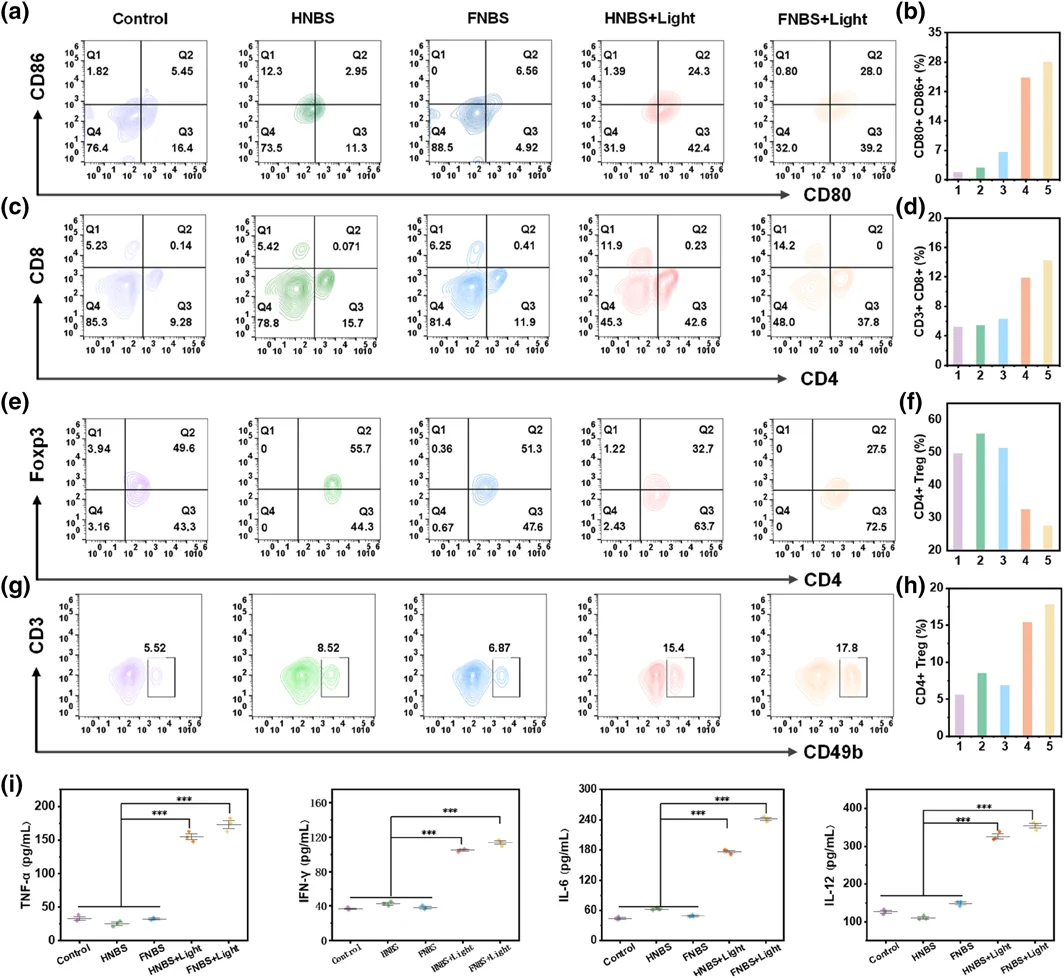
Systemic antitumor immune response by FNBS. (a) Representative flow cytometry plots and (b) quantification of mature dendritic cells (DCs) in primary tumors (CD11c^+^ CD80^+^ CD86^+^). (c) Representative flow cytometry plots and (d) quantification of T helper cells (CD3^+^ CD4^+^) and cytotoxic T lymphocytes (CTLs, CD3^+^ CD8^+^) in primary tumors from mice after different treatments. (e) Representative flow cytometry plots and (f) quantification of regulatory T cells (Tregs). (g) Representative flow cytometry plots and (h) percentages of natural killer (NK) cells (CD3^−^ CD49b^+^). (i) Levels of TNF‐α, IFN‐γ, IL‐6, and IL‐12 in mice at 7 days after different treatments. Results are presented as mean ± SD (*n* = 3). Statistical analysis was performed using one‐way ANOVA (**p < 0.01, and ***p < 0.001).

Given that DCs serve as the most potent antigen‐presenting cells and represent a critical link between innate and adaptive immunity, the impact of FNBS‐induced ICD on DC maturation and subsequent T cell activation was further investigated During ICD, DC maturation can be induced by DAMPs. The following maturation, DCs process and present tumor antigens to T cells, thereby accelerating T cell activation and proliferation. Accordingly, flow cytometry analysis was conducted to evaluate DC maturation (CD11c^+^, CD80^+^, CD86^+^) [38] and T cell activation, including T helper cells (CD3^+^CD4^+^) and cytotoxic T lymphocytes (CTLs, CD3^+^CD8^+^)[39]. As shown in Figure 7a,b, the proportion of CD80^+^CD86^+^ cells in primary tumors reached 28.0% in the FNBS + light group, which was approximately fivefold higher than that in the control group, indicating enhanced DC maturation. Concomitantly, adaptive immune activation was evidenced by significantly increased infiltration of CD3^+^CD4^+^ T helper cells and CD3^+^CD8^+^ CTLs within primary tumors. The highest proportions of infiltrating CD3^+^CD4^+^ and CD3^+^CD8^+^ T cells were observed in the FNBS+light group, which were 4.0‐ and 2.7‐fold higher than those in the control group, respectively. In parallel, regulatory T cells (Tregs, CD4^+^CD25^+^Foxp3^+^), which are the principal immunosuppressive T lymphocytes recruited by tumors to inhibit CTL activity, were analyzed [40]. In the control group, intratumoral Tregs accounted for up to 49.6% of the primary tumor immune population (Figure 7e,f), confirming the highly immunosuppressive tumor microenvironment characteristic of “cold” 4T1 tumors.

Besides, the proportion of intratumoral Tregs in the FNBS + light group markedly decreased to 27.5%, indicating alleviation of immunosuppression by oncolytic pyroptosis. Furthermore, the proportion of intratumoral NK cells (CD3^−^CD49b^+^) increased from 5.5% in the control group to 17.8% following FNBS + light treatment, suggesting that proinflammatory pyroptosis concurrently activates innate immunity. Collectively, these results demonstrate that FNBS‐mediated PIT not only eradicates tumor cells via ROS‐induced pyroptosis but also robustly activates antitumor immune responses by promoting DC maturation, enhancing T cell and NK cell infiltration, and suppressing Treg accumulation. Compared with prior furan‐mediated photopolymerization RNAi therapy [41], this work integrates furan‐mediated photopolymerization with a novel high‐performance photosensitizer to design FNBS. It operates independently of tumor alkaline phosphatase expression and efficiently suppresses tumor proliferation under both normoxic and hypoxic conditions—even at ultralow light doses.

## 3 CONCLUSION

This study reports the rational design and development of FNBS, a red‐light‐activated small‐molecule photosensitizer that integrates PIT and RNAi, thereby overcoming key limitations associated with nanocarrier‐based combination therapies. In vitro studies demonstrate that FNBS retains the favorable photophysical properties and intrinsic lysosomal targeting capability of phenothiazine dyes. Upon 660 nm light irradiation, FNBS exerts dual synergistic functions: on one hand, photoinduced ROS generation triggers lysosomal escape and activates the canonical NLRP3 inflammasome—caspase‐1—GSDMD pathway, leading to pyroptosis and subsequent ICD; on the other hand, ROS‐mediated oxidation of the furan moiety enables RNA crosslinking and degradation, resulting in effective RNA interference. RNA‐seq analysis further links FNBS‐induced cell death to disrupted RNA metabolism. In vivo, FNBS exhibits prolonged tumor retention for up to 72 h and, upon irradiation, achieves potent inhibition of both primary and distant tumors, with inhabitation rates of 90.2% and 93.9%, respectively, through remodeling the tumor microenvironment. FNBS‐induced pyroptosis and ICD promote robust immune activation, as evidenced by enhanced DC maturation (5.0‐fold increase vs. control), increased infiltration of T helper cells (4.0‐fold) and CTLs (2.7‐fold), and effective conversion of immunologically “cold” tumors into “hot” tumors. Importantly, FNBS also displays excellent biocompatibility in vivo. Compared with conventional nanocarrier‐based systems, FNBS avoids issues such as low drug‐loading efficiency and complex synthesis, offering a carrier‐free, small‐molecule approach for multimodal cancer therapy. Overall, this work demonstrates the feasibility of integrating PIT and RNAi via rational molecular design, providing a promising and clinically viable paradigm for the development of efficient, safe, and multifunctional antitumor agents.

## CONFLICT OF INTEREST STATEMENT

The authors declare no competing financial interests or personal relationships that could have influenced this work. Jiangli Fan is an editorial board member of *Smart Molecules* and co‐author of this article. They were excluded from editorial decision‐making related to the acceptance of this article for publication in the journal.

## ETHICS STATEMENT

All protocols for animal studies conformed to the Guide for the Care and Use of Laboratory Animals and approved by the Dalian University of Technology Animal Care and Use Committee (DUTNI250312‐03).

## Supporting information

Supporting Information S1

## Data Availability

The data that supports the findings of this study are available in the supplementary material of this article.
