# Supplementary material for: A photoimmune synergizing RNA interference technology for highly effective antitumor treatment
Source: Smart Mol. 2026 Aug 23:e70095. Online ahead of print. doi: 10.1002/smo2.70095 (PMC13501089; doi:10.1002/smo2.70095)
Supplement: Supplementary file 1 [file SMO2-9999-0-s001.docx]

**A Photoimmune Synergizing RNA Interference Technology for Highly Effective Antitumor Treatment**

Xiang Xia, Bingbing Wu, Xueze Zhao, Wen Sun, Jianjun Du^*^, Jiangli Fan^*^, Xiaojun Peng

Xiang Xia, Bingbing Wu, Xueze Zhao, Wen Sun, Jianjun Du, Jiangli Fan, Xiaojun Peng

State Key Laboratory of Fine Chemicals, Frontiers Science Center for Smart Materials-Oriented Chemical Engineering

Dalian University of Technology

2 Linggong Road, Hi-tech Zone, Dalian 116024, China

E-mail: dujj@dlut.edu.cn, fanjl@dlut.edu.cn.

Xiang Xia

The Key Laboratory of Laboratory Medicine, Ministry of Education, School of Laboratory Medicine and Life Sciences

Wenzhou Medical University

Wenzhou Medical College Chashan University, Town Ouhai District, Wenzhou, 325035, China.

**Materials and Methods**

Materials：

(1) Chemical reagents

*N,N*-diethylp-phenylene diamine, aluminum sulfate, sodium thiosulfate, zinc chloride, potassium dichroate, 3-iodopropionic acid, triethylamine, HOBt, EDCI, 1,3-diphenylisobenzofuran (DPBF) were purchased from Energy Chemical. 1,2,3,4-tetrahydro-1-naphthylamine, furan-2-ylmethylamine were purchased from Bide Pharmatech.

(2) Biological reagents

Thiazol blue (for MTT assay) was purchased from Energy Chemical. Annexin V-FITC apoptosis detection kit (C1062S), Calcein/PI cell activity and cytotoxicity detection kit (C2015S), Mito-Tracker Green (mitochondrial green fluorescent probe) (C1048), Lyso-Tracker Green (lysosome green fluorescent probe) (C1047S), Hoechst 33258 (C1011), and Lipid Droplet Green Fluorescent Detection kit (BODIPY 493/503) (C2053S), RNAeasy^TM^ Animal RNA Extraction Kit (centrifugal column) (R0026), BeyoGel™ agarose preset gel (2%, NA-Red, TAE, 8 Wells) (D0163S), acridine orange staining kit (C0233S), lactate dehydrogenase cytotoxicity assay kit (C0016), ATP assay kit (S0026B), HMGB1 Rabbit Monoclonal Antibody (AG2167), Calreticulin Rabbit Monoclonal Antibody (AF1666), DAPI (C1002), GAPDH Mouse Monoclonal Antibody (AF2819-50μL), Alexa Fluor 488 labeled goat anti-rabbit IgG(H+L) (A0423), BeyoColor™ molecular weight standard (15-120kD) (P0078), BCA Protein concentration Determination kit (P0012), horseradish peroxidase labeled goat anti-rabbit IgG(H+L) (A0208), BeyoECL Plus (Hypersensitive ECL chemiluminescence Kit) (P0018S) was purchased from Beyotime Biotechnology. ELISA kit IL-18 (mouse source) and IL-1β (mouse source) were purchased from Shanghai Enzyme-linked Biotechnology. Anti-GSDMD antibody (Abcam, ab219800) and anti-pro-Caspase-1 antibody (Abcam, ab179515) were purchased from Abcam. APC Anti-Mouse CD3 Antibody[17A2], PE Anti-Mouse CD8a Antibody[53-6.7], FITC Anti-Mouse CD4 Antibody[GK1.5], FITC Anti-Mouse CD11c Antibody[N418], APC Anti-Mouse CD80 Antibody[16-10A1], and PE Anti-Mouse CD86 Antibody[GL-1] were purchased from Elabscience. Hyaluronidase and collagenase IV were purchased from Solarbio Science&Technology.

(3) Cell lines and cell culture medium

Murine breast cancer cells (4T1), human breast cancer cells (MDA-MB-231) and human hepatoma cells (HepG2) were purchased from National Collection of Authenticated Cell Cultures. Cell culture medium DMEM, phosphate buffer PBS, and bovine serum albumin FBS were purchased from Thermo Fisher Scientific Co., LTD. Trypsin cell digest (0.25% trypsin, containing phenol red) (C0203-100mL) was purchased from Beyotime Biotechnology.

(4) Instruments and equipment

NMR imaging was performed using Bruker Avance II at 400 MHz to obtain ^1^HNMR and ^13^CNMR, with tetramethyl silicon as the internal standard for the chemical shift, δ. Data from high-resolution mass spectrometry (HRMS) were measured using TOF LC/MS (G6224A) and LTQ Orbitrap XL. The UV-vis absorption and fluorescence spectra were tested by Duetta UV fluorescence integrated machine. Fluorescence images of cells and sections were obtained by confocal photography with an Olympus FV3000 laser. The absorbance of each well of 96-well plate was obtained by Varioskan LUX Multimode Microplate Reader for cell MTT assay and WB protein like BCA concentration determination. Tissue sections were performed by Leica CM1860 UV cry sectioning mechanism. Flow analysis was performed using Attune^TM^ NxT, Acoustic Focusing Cytometer Model: AFC2. The multi-functional image workstation GelView 6000ProII was used for gel electrophoresis imaging and small animal imaging and analysis.

**Synthesis methods**

Figure S1. Synthesis of HNBS and **FNBS**

**Compound 1**: *N,N*-Diethyl-p-phenylenediamine (1 g, 6.09 mmol) was added to a stirred aqueous solution (10 mL) containing aluminum sulfate (4.11 g, 6.52 mmol). Sodium thiosulfate (2.21 g, 14 mmol) and zinc chloride (0.872 g, 6.39 mmol) were subsequently introduced into the mixture. The reaction mixture was then cooled in an ice bath, and a slow addition of 4 mL of potassium dichromate aqueous solution (0.49 g, 1.68 mmol) was performed. The mixture was maintained under stirring in the ice bath for a duration of 2 h; during this time, a thick precipitate formed which was subsequently filtered and washed with acetone. The crude product obtained was refluxed in methanol (12 mL), followed by filtration to yield a dark gray solid weighing 0.79 g, corresponding to a yield of 47%. The resulting product can be utilized without further purification.

**Compound 2**: Reflux 1,2,3,4-tetrahydro-1-naphthylamine (0.5 g, 3.4 mmol) with 3-isopropionic acid (0.68 g, 3.4 mmol) and triethylamine (0.69 g, 6.8 mmol) in tetrahydrofuran (4 mL) at a temperature of 66 °C for a duration of 18 h. The reaction mixture was subsequently cooled to room temperature and filtered. Following dilution with dichloromethane and washing with water, the aqueous layer was removed by drying over anhydrous sodium sulfate. The product was then separated and purified using silica gel column chromatography with a mobile phase consisting of MeOH/DCM in a ratio of 50/1 to yield product **Compound 2** (150 mg, 0.68 mmol, 20%). ^1^H NMR (400 MHz, Methanol-d_4_) δ 6.94 (t, *J* = 7.8 Hz, 1H), 6.48 (d, *J* = 8.0 Hz, 1H), 6.44 (dd, *J* = 7.6, 1.1 Hz, 1H), 3.41 (t, *J* = 6.7 Hz, 2H), 2.69 (t, *J* = 6.2 Hz, 2H), 2.61 (t, *J* = 6.7 Hz, 2H), 2.41 (t, *J* = 6.5 Hz, 2H), 1.88 – 1.81 (m, 2H), 1.76 – 1.69 (m, 2H). HRMS: m/z calculated for (C_13_H_17_NO_2_, [M-H]^-^): 218.1259; found: 218.1179.

**Compound 3**: **Compound 2** (113 mg, 0.515 mmol), HOBt (83.5 mg, 0.618 mmol), EDCI (95.87 mg, 0.618 mmol), and triethylamine (156.5 mg, 1.546 mmol) were dissolved in 50 mL of dichloromethane at room temperature. After stirring for 30 minutes, fur-2-methylamine (50 mg, 0.515 mmol) was added to the solution and stirred at room temperature for an additional 12 h. Following the reaction, the product **Compound 3** (90 mg, 0.30 mmol) was obtained by column chromatography on silica gel with a yield of 58.6%. ^1^H NMR (400 MHz, Chloroform-d) δ 7.37 (s, 1H), 7.34 (s, 1H), 7.03 (t, *J* = 7.8 Hz, 1H), 6.54 (d, *J* = 7.6 Hz, 1H), 6.50 (d, *J* = 8.0 Hz, 1H), 6.33 (s, 1H), 5.89 (s, 1H), 4.27 (d, *J* = 5.5 Hz, 2H), 3.51 (t, *J* = 6.0 Hz, 2H), 2.73 (t, *J* = 6.2 Hz, 2H), 2.54 (t, *J* = 6.0 Hz, 2H), 2.37 (t, *J* = 6.4 Hz, 2H), 1.84 (p, *J* = 6.2 Hz, 2H), 1.74 (h, *J* = 5.8 Hz, 2H). HRMS: m/z calculated for (C_18_H_22_N_2_O_2_, [M+H]^+^): 299.1681; found: 299.1760.

**HNBS**: HNBS was synthesized as described in our earlier report [1].

**FNBS**: **Compound 3** (85 mg, 0.285 mmol) and **Compound 1** (158 mg, 0.57 mmol) were dissolved in a methanol solution (30 mL). The reaction mixture was heated to 80 °C, after which Ag_2_CO_3_ (156 mg, 0.57 mmol) was added and the mixture was refluxed for 30 minutes. Upon cooling to room temperature, Ag_2_CO_3_ and excess **Compound 1** were removed by filtration. Methanol was subsequently eliminated through distillation under reduced pressure, yielding a blue solid (30 mg, 0.06 mmol), which was obtained via silica gel column chromatography with DCM/MeOH as the eluent at a ratio of 50/1 (V/V), resulting in an overall yield of 21.6%. ^1^H NMR (400 MHz, Methanol-d_4_) δ 7.98 (d, *J* = 9.6 Hz, 1H), 7.46 (dd, *J* = 9.6, 2.8 Hz, 1H), 7.38 (t, *J* = 1.2 Hz, 1H), 7.35 (t, *J* = 1.8 Hz, 1H), 7.33 (d, *J* = 2.8 Hz, 1H), 7.22 (s, 1H), 6.33 – 6.30 (m, 1H), 4.21 (s, 2H), 3.83 (t, *J* = 6.6 Hz, 2H), 3.75 (q, *J* = 7.1 Hz, 4H), 3.22 (t, *J* = 6.1 Hz, 2H), 2.65 (t, *J* = 6.6 Hz, 2H), 2.52 (t, *J* = 6.2 Hz, 2H), 2.00 – 1.85 (m, 4H), 1.36 (t, *J* = 7.1 Hz, 6H). HRMS: m/z calculated for [C_28_H_33_N_4_O_2_S]^+^: 489.2319; found: 489.2270.

**Absorption and fluorescence measurements**

After vacuum drying the dye, the samples were accurately weighed and prepared as a 10 mmol/L DMSO solution. They were then stored in a refrigerator at 0-4 °C until required for use. For analytical characterization, an aliquot of the mother liquor was transferred to a brown volumetric flask, completely dissolved, and mixed with appropriate reagents to prepare a 10 μM dye solution. UV-Vis absorption and fluorescence emission spectra were subsequently recorded, with all measurements conducted at room temperature.

**Singlet oxygen (^1^O_2_) detection.**

Adding a specific number of compounds (FNBS and MB) to the cuvette containing a methanol solution was performed to achieve an absorbance of approximately 0.3 at the maximum absorption wavelength. Subsequently, a certain quantity of DPBF was added to all three cuvettes to attain an absorbance of about 1.0 at 415 nm. Following this preparation, a laser emitting light at 660 nm (with an intensity of 1.5 mW/cm²) was utilized to irradiate the cuvette plate in intervals of 5 seconds while measuring the absorption spectrum concurrently. The efficiency of singlet oxygen (^1^O_2_) production post-photoexcitation of the compound was evaluated by comparing the rate of absorbance reduction among different groups containing DPBF. The quantum yield for ^1^O_2_ can be calculated using the following formula (1):

$\emptyset_{\Delta sam}=\emptyset_{\Delta std}(\frac{m_{sam}}{m_{std}})(\frac{F_{std}}{F_{sam}})$	(1)

"sam" and "std" denote the sample under investigation and the reference sample (MB), respectively. The term "m" refers to the slope of the decline in DPBF absorbance at a wavelength of 415 nm, where F is defined as 1-10^-O.D.^, with O.D. representing the absorbance of the sample at its maximum absorption wavelength.

**Superoxide radical (O_2_^−•^) detection**

A 1×1×3 cm cuvette was filled with an aqueous solution containing 5 μM FNBS and 10 μM DHE. The cuvette was subsequently exposed to monochromatic light at a wavelength of 660 nm (3 mW/cm^2^). Fluorescence spectra were recorded at two-minute intervals, utilizing an excitation wavelength of 535 nm and an emission wavelength range of 545-640 nm.

**Hydroxyl radical (•OH) detection.**

A 1×1×3 cm cuvette was filled with an aqueous solution containing 5 μM FNBS and 10 μM HPF. The cuvette was then exposed to monochromatic light at a wavelength of 660 nm (3 mW/cm^2^). The corresponding fluorescence spectra were recorded with an excitation wavelength of 488 nm and an emission wavelength range of 490-650 nm.

**Cell Culture.**

4T1 murine breast cancer cells, MDA-MB-231 human breast cancer cells, and HepG2 human hepatocellular carcinoma cells were maintained in DMEM supplemented with 10% fetal bovine serum (FBS), 1% penicillin (100 U/mL), and 1% streptomycin (100 μg/mL). Cells were incubated at 37 °C under standard culture conditions (21% O_2_ and 5% CO_2_) until reaching logarithmic growth phase. Following trypsinization, cell suspensions were prepared for subsequent experiments.

**Cellular uptake assay**

Cells were seeded into confocal microscopy dishes at optimal density and allowed to adhere for 24 hours under standard culture conditions. Prior to treatment, adherent cells were gently washed twice with phosphate-buffered saline (PBS). A fluorescent dye solution (1 μM final concentration in complete medium) was applied to cells. Fluorescence signals were detected using a confocal laser scanning microscopy (CLSM) system with excitation at 635 nm and emission collection between 650-750 nm.

**Investigation into the Mechanism of RNA Cross-Linking**

(1) RNA extraction

1. Cell collection

Adherent cells at densities of 5×10^5^ to 1×10^6^ were collected. After removal of the medium, 300 μL lysis buffer was added to the culture vessel, and the cell mass was fully lysed by gentle blowing for 5-10 times until the suspension was homogeneous and clear, and then transferred to a sterile centrifuge tube.

2. Binding reaction

Binding buffer was added to a medium volume of the lysate, and the mixture was gently turned over 3-5 times. Flocculent precipitation may occur at this stage, which is a normal phenomenon.

3. Purification treatment

The mixture (including the precipitate) was transferred to an RNA purification column, centrifuged at 12,000 ×g for 30 s, and the waste solution was discarded. Do the following in turn: 600 μL washing buffer I was added, centrifuged at 12,000×g for 30 seconds, and the solution was discarded. 600 μL of washing buffer II was added, centrifuged at 12,000 ×g for 30 s, and the solution was discarded. Repeat the washing buffer II step once; Residual liquid was thoroughly removed by centrifugation at 16,000 ×g for 2 min.

4. RNA elution

The purification column was transferred to a special elution tube, 30-50 μL of elution buffer was added, left at room temperature for 2-3 minutes, and centrifuged at maximum speed for 30 seconds. The liquid in the collection tube was a high-purity RNA solution.

(2) Agarose gel electrophoresis analysis

FNBS and RNA cross-linking experiment: the above extracted RNA was divided into four groups: RNA group, RNA+Light group, RNA+FNBS group, and RNA+FNBS+Light group. The concentration of FNBS was 1 μM, and the light condition was 660 nm, 2 mW/cm^2^, and 5 min. The samples were then subjected to electrophoresis (150 V) by adding the agarose preset gel until the bromophenol blue band reached the middle and lower part of the gel plate. Subsequently, gel electrophoresis imaging (546 nm (EB) and 632 nm (Cy7)) was performed.

Gel electrophoresis imaging of FNBS-degraded RNA: The above extracted RNA was mixed with different concentrations of FNBS (0, 1, 2, 3, 4, and 5 μM) and illuminated (660 nm, 2 mW/cm^2^, 5 min), and gel electrophoresis was performed as described above.

(3) determination of nucleic acid concentration

4T1 cells were seeded in six-well plates and washed twice with PBS until the cells had grown to 80 to 90% density. Three groups of Control, FNBS, and FNBS+Light were set up, and the concentration of FNBS was 1 μM. After illumination (660 nm, 2 mW/cm^2^, 5 min), RNA was extracted from cells in each group. RNA concentration was also calculated.

**Intracellular detection of ROS**

4T1 cells were seeded in confocal dishes at 1×10^5^ cells/cm^2^ and cultured for 24 h. After three PBS washes, cells were incubated with 2',7'-dichlorofluorescein diacetate (DCFH-DA; 1:2000 dilution) and the photosensitizer FNBS (1 μM) for 30 min. Fluorescence imaging was performed using a confocal microscope (λ_ex_: 488 nm, λ_em_: 495-540 nm) following 1 min irradiation with a 660 nm LED light source (2 mW/cm^2^).

**Localization test of FNBS in cells**

4T1 cells in logarithmic phase were co-incubated with 1 μM FNBS and organelle-specific probes: Lyso Tracker Green (lysosomes)、Mito Tracker Green (mitochondria)、BODIPY 493/503 (lipid droplets)、Hoechst 33258 (nucleus). After 30 min incubation, colocalization analysis was conducted using CLSM with sequential excitation. (Lyso/Mito/LDs λ_ex_: 488 nm, λ_em_: 495-610 nm; Hoechst33258 λ_ex_: 405 nm, λ_em_: 420-550 nm; FNBS λ_ex_: 635 nm, λ_em_: 650-750 nm).

**Cytotoxicity assessment**

(1) Normoxic conditions

4T1, HepG2, and MDA-MB-231 cells were seeded in 96-well plates (1×10^4^ cells/well) and cultured to ~60% confluence. Cells were treated with FNBS (graded concentrations, the final volume fraction of DMSO in the assay system was carefully maintained at ≤0.005) for 30 min, followed by 660 nm LED irradiation (1.5 mW/cm^2^, 5 min). Parallel dark controls received identical treatments without irradiation. After 24 h incubation, cell viability was assessed via MTT assay: 0.5 mg/mL MTT in DMEM (100 μL/well, 4 h at 37 °C), followed by formazan dissolution in DMSO (100 μL) and absorbance measurement at 490 nm.

(2) Hypoxic Conditions

Cells were preconditioned in a hypoxic chamber (4% O_2_, 4 h) prior to FNBS treatment. Subsequent procedures followed the normoxic protocol, using enhanced irradiation intensity (5 mW/cm^2^). All experimental controls maintained identical hypoxic conditions throughout.

$cell viability=\frac{{OD}_{test group}-{OD}_{control group}}{{OD}_{0 group}-{OD}_{control group}}\times100\%$	(2)

In the formula, OD_test group_ is the absorbance of each group detected, OD_control group_ is the absorbance of blank control group, and OD_0 group_ is the absorbance when the drug concentration is 0 under the corresponding treatment.

**Live/Dead Cell Staining**

4T1 cells were cultured to optimal confluence and divided into four treatment groups:

Control (untreated)、Light (660 nm, 3 mW/cm^2^, 10 min)、FNBS (1 μM)、FNBS+Light (1 μM + irradiation). Following two PBS washes, experimental groups received designated treatments. After 24 h incubation, cells were stained with Calcein-AM/PI using manufacturer's protocol: Calcein-AM (viability marker): λ_ex_ 488 nm / λ_em_ 500-540 nm; Propidium iodide (PI, necrotic marker): λ_ex_ 535 nm / λ_em_ 550-620 nm. Confocal imaging was performed immediately post-staining.

**Flow Cytometry Analysis**

4T1 cells in 6-well plates (2×10^5^ cells/well) were treated with 1 μM FNBS for 30 min, then irradiated (660 nm, 2 mW/cm^2^, 10 min). After 2 h additional incubation, cells were processed using Annexin V-FITC/PI Apoptosis Kit: Harvested by gentle trypsinization; Washed with cold PBS; Resuspended in 100 μL binding buffer; Stained with 5 μL Annexin V-FITC and 10 μL PI (15 min, dark). Then flow cytometry was used for up-machine detection.

**Lysosomal Damage Assay**

To elucidate the mechanism underlying FNBS-mediated lysosomal destruction, acridine orange (AO) was employed as an indicator of lysosomal integrity. AO is a protonated oligomeric compound that binds to single-stranded DNA or RNA; specifically, one molecule of AO interacts with a single phosphate group and exhibits red fluorescence. 4T1 cells were seeded in confocal culture dishes. Upon reaching a specific density, the cells were washed twice with PBS and subsequently divided into Control, Light, FNBS, and FNBS+light groups. Dishes containing FNBS (1 μM) were incubated for 30 min before being illuminated at 660 nm with an intensity of 2 mW/cm^2^ for 5 min. After allowing for a designated period post-illumination, the confocal dish was washed twice with PBS. Acridine orange solution was then added to the dishes and incubated at 37 °C for 2 to 10 min. Following incubation, the staining solution was removed by aspiration and the wells were washed twice with PBS. An appropriate volume of cell culture medium was added to cover the bottom of each well plate prior to observation under a confocal microscope. (excitation wavelength: 488 nm; Green fluorescence: emission wavelength, 500-520 nm; Red fluorescence: emission wavelength, 530-640 nm)

**LDH and ATP Release Assays**

4T1 cells were seeded in 96-well plates, with each well containing 100 μL of DMEM. Once the cells reached approximately 80% confluence, various concentration gradients of FNBS (0, 20, 40, 80, 160, and 320 nM) were established. Following a half-hour incubation period with the cells, they were exposed to light at a wavelength of 660 nm and an intensity of 2 mW/cm^2^ for a duration of 5 min. Subsequently, the cell supernatant was collected after allowing it to rest for a specified period. The LDH activity in the supernatants from each group was measured according to the instructions provided by the LDH Cytotoxicity Assay Kit.

For the ATP release assay: after completing identical treatments on the cells, the supernatant was discarded and replaced with cell lysis buffer which was then incubated on ice to facilitate lysis. Afterward, centrifugation at 12,000 g for five minutes at 4 °C was performed to remove debris from lysed cells; this clarified supernatant was utilized for subsequent assays. Finally, ATP content within these samples was quantified following guidelines outlined in the ATP Assay Kit's instructions.

**Immunofluorescence Staining Experiments**

The levels of HMGB1 and CRT in 4T1 cells subjected to various treatments were assessed through immunofluorescence staining. Cells treated with different conditions (Control, Light, FNBS, FNBS+Light) were either exposed to laser light for 5 min or shielded from light. Following treatment, the cells were cultured for an additional 2 h, washed three times with PBS, and fixed using an immunostaining fixative at room temperature for 20 min. After washing three more times with PBS, the cells were blocked with an immunostaining blocking solution for 2 h. Subsequently, they underwent three additional washes with PBS. Next, the cells were incubated overnight at 4 °C with a rabbit monoclonal antibody specific to HMGB1/CRT. After this incubation period, the cells were washed three times with PBS and then stained for one hour at room temperature in the dark using Alexa Fluor 488-labeled Goat Anti-Rabbit IgG(H+L). The final step involved incubating the cells with DAPI staining solution for 10 minutes. CLSM images were acquired under the following parameters: DAPI channel: E_x_ = 405 nm; E_m_ = 430-470 nm; Alexa Fluor 488 channel: E_x_ = 488 nm; E_m_ = 500-550 nm.

**Western blotting analysis.**

4T1 cells were seeded in six-well plates at a density of 1×10^5^ cells per well. Once the cell density reached approximately 85%, FNBS (1 μM) was added and incubated for 30 minutes, followed by exposure to light at a wavelength of 660 nm with an intensity of 2 mW/cm^2^ for 5 min. Upon observing pyroptosis in a greater number of cells, the cultures were washed twice with PBS and subsequently lysed by adding cell lysis buffer. After lysis, the supernatant was centrifuged at 4 °C, and the histone content was quantified using a standard protein solution. Following this step, protein loading buffer was added to the samples, which were then boiled at 95 °C for 7 min before being stored in a freezer for further analysis.

5 μL of marker and various experimental groups were loaded into the wells of a preformed PAGE gel. Electrophoresis was subsequently halted when bromophenol blue approached the bottom of the gel. The PVDF membrane was activated using methanol, and the gel electrophoresis apparatus was placed in an ice bath for membrane transfer (300 mA, 70 min). Upon completion, the PVDF membrane was washed with TBST for five cycles of six minutes each. A western blocking solution was then applied to block non-specific binding sites for one hour, followed by another wash step. The primary antibody was added and incubated at 4 °C overnight; after washing, the secondary antibody was incubated at 37 °C. Following further washes, a chemiluminescence development solution was added, and imaging was performed using a gel electrophoresis imager.

**Establishment of Tumor Model (Subcutaneous 4T1 Breast Cancer)**

**Ethics Statement**

All protocols for animal studies conformed to the Guide for the Care and Use of Laboratory Animals and approved by the Dalian University of Technology Animal Care and Use Committee (DUTNI250312-03).

In this study, six-week-old female BALB/c mice were obtained from Liaoning Changsheng Biotechnology Co., Ltd. All experimental procedures were conducted in accordance with the Guide for the Care and Use of Laboratory Animals as well as the regulations set forth by the National Research Council. The protocols received approval from the Animal Care and Use Committee at Dalian University of Technology. A subcutaneous tumor model of breast cancer was established in these mice by injecting 100 μL of a 1×10^6^ cell suspension containing 4T1 cells into the right axillary region. Once the tumor volume reached approximately 200 mm^3^, the mice were utilized for in vivo imaging and photodynamic therapy experiments.

**Fluorescence Imaging in Mice**

FNBS (100 μM, 100 μL) was administered via intratumoral injection into mice bearing 4T1 tumors, as previously described. Fluorescence imaging was conducted using a small animal imager at various time points. The fluorescence intensity was analyzed to ascertain the time of maximal FNBS accumulation within the tumor, thereby determining the optimal timing for treatment.

**In vivo Tumor Photodynamic Therapy**

Bilateral tumor models were initially established by subcutaneously injecting 4T1 tumor cells into the right side of the mice. Five days later, an additional injection of 4T1 tumor cells was administered to the left side of the mice. Treatment commenced four days following this second injection. Mice were randomly assigned to five groups (n = 5 each), which included: (1) Intratumoral saline administration (100 μL) as the Control group; (2) Injection of HNBS (100 μM, 100 μL. DMSO percent was 10%) via the intratumoral (HNBS group); (3) Intratumoral injection of FNBS (100 μM, 100 μL. DMSO percent was 10%) (FNBS group); (4) Intratumoral injection of HNBS (10 nM) followed by exposure to a 660 nm laser at an intensity of 6J/cm^2^ after a half-hour interval (HNBS+Light group) and Intratumoral injection of FNBS (10 nM) followed by exposure to a 660 nm laser at an intensity of 6J/cm2 after a half-hour interval (FNBS+Light group). Body weight and tumor volume in mice were measured every other day, for 14 days.

**In vivo anti-tumor immunity effect**

The primary tumors, distant tumors, and spleens were harvested from each group of mice following photodynamic therapy (PDT). The primary tumor and distant tumor samples were excised and digested using digestive enzymes. Subsequently, the mixture was filtered through a 200-mesh filter, centrifuged to discard the supernatant, and washed with DMEM. After another round of centrifugation to remove the supernatant, red blood cell lysate was added and incubated at 37 °C for 5 minutes. Medium was then introduced to aspirate the supernatant before adding face buffer for an additional wash step; finally, the supernatant was discarded. Following this preparation, CD8^+^, CD3^+^and CD4^+^ antibody solutions were added to the cells for staining over a period of 30 minutes. The cells were then centrifuged again to discard the supernatant before introducing 500 μL of 1% tissue fixative in preparation for flow cytometry analysis. For spleen samples, they were directly ground using PBS, omitting the digestion step while maintaining consistency with other procedures; however, antibodies used in this case were switched to CD86^+^, CD80^+^ and CD11c^+^.

**In Vivo Biosafety Evaluation**

(1) Hemolysis Test

Fresh sterile material was defibrinated. A total of 3 mL of sheep blood was added to 6 mL of PBS buffer and centrifuged at 1500 rpm for 15 min. The plasma and brown precipitate were removed, and the red blood cells were collected and washed several times with PBS until no red color was observed in the supernatant. The obtained red blood cells were then suspended in 200 μL of PBS and diluted with an additional 9.8 mL of PBS. Subsequently, 0.5 mL of this red blood cell suspension was mixed with 0.5 mL of PBS containing varying concentrations of FNBS, while a mixture of red blood cells with ultra-pure water served as the control group (100% hemolysis). The samples were incubated at 37 °C on a shaker set to 180 rpm for three hours before being centrifuged at 1500 rpm for eight minutes. The absorbance of the supernatant was measured using a microplate reader, from which the hemolysis rate was calculated.

$Hemolysis rate=\frac{{OD}_{test group}-{OD}_{negative control group}}{{OD}_{positive control group}-{OD}_{negative control group}}\times100\%$	(3)

(2) Whole Blood Analysis

Healthy mice were divided into four groups (n = 5), including: 1. Control, 2. HNBS, 3. FNBS, 4. HNBS+light, 5. FNBS+light. Whole blood samples were collected for analysis. The normal saline group served as a negative control.

(3) Hematoxylin and eosin (H&E) staining

After PDT, the major organs (heart, liver, spleen, lung and kidney) and tumors were collected for histological analysis by hematoxylin and eosin (H&E) staining.

**Statistical analysis.**

All the experiments were replicated and the specific number of replicates was either annotated in the figure captions or specific sections. Unless otherwise specified, statistical significance was determined by the unpaired, two-tailed Student’s t-test at ****p < 0.0001, ***p < 0.001, **p <0.01 and *p < 0.05. Data were presented as mean ± standard deviation (SD).

**Figure S2**. Absorption and emission spectra of HNBS and FNBS in different solvents

**Figure S3**. Intracellular ^1^O_2_ detection using DPBF as detector.

**Figure S4**. Intracellular •OH and O_2_^•−^ detection using HPF (Ex: 488 nm, Em: 500-540 nm)and DHE (Ex: 561 nm, Em: 590–620 nm) as fluorescent probe

**Figure S5**. Raw images of WB of GSDMD, GADPH, Caspase-1 and NLRP3 and quantitative analysis

**Figure S6**. Sample correlation heatmap of FNBS and FNBS+light

**Table S1.** RNA concentration of groups with different treatment in vitro

**Figure S7**. Fluorescence imaging of FNBS with intratumoral injection.

**Figure S8**. Fluorescence imaging of HNBS with intratumoral injection.

**Figure S9.** Hemolysis rates of red blood cells (RBCs) treated with different concentrations of FNBS

**Figure S10**. H&E staining of organs and tumor tissues from the tumor-bearing mice treated by different treatment.

**Figure S11**. The blood routine examinations of mice were performed after different treatments. White blood cells (WBC), Red blood cells (RBC), concentration of hemoglobin (HGB), hematocrit (HCT), mean corpuscular volume, RBC volume distribution width (RDW), mean corpuscular hemoglobin concentration, and mean corpuscular hemoglobin (MCH).

**Figure S12**. Immunofluorescent staining images of Caspase-1, GSDMD, CRT, and HMGB-1 of the primary tumor tissues excised from the mice in each group. Scale bar is 100 μm.

**Figure S13**. ^1^H NMR spectrum of compound 2 in MeOD.

**Figure S14**. ^13^C NMR spectrum of compound 2 in MeOD.

**Figure S15**. ^1^H NMR spectrum of compound 3 in CDCl_3_.

**Figure S16**. ^13^C NMR spectrum of compound 3 in CDCl_3_.

**Figure S17**. ^1^H NMR spectrum of FNBS in MeOD.

**Figure S18**. ^13^C NMR spectrum of FNBS in MeOD.

**Figure S19**. HRMS spectrum of compound 2.

**Figure S20**. HRMS spectrum of compound 3.

**Figure S21**. HRMS spectrum of FNBS.

Figure S22 ^1^H NMR spectrum of HNBS in MeOD.

Figure S23. HRMS spectrum of HNBS.

Reference:

1. X. Xia, R. Wang, Y. Hu, S. Long, W. Sun, J. Fan, X. Peng, Nonconjugated Structural Distortion Promoting the Formation of NIR Triplet States in Phenothiazine Dyes for Cancer Photoimmunotherapy, Angew. Chem. Int. Ed. 2025, 64, e202507157.
